# Dietary nutrients during gestation cause obesity and related metabolic changes by altering DNA methylation in the offspring

**DOI:** 10.3389/fendo.2024.1287255

**Published:** 2024-02-20

**Authors:** Szilvia Bokor, Ildikó Csölle, Regina Felső, Réka A. Vass, Simone Funke, Tibor Ertl, Dénes Molnár

**Affiliations:** ^1^ Department of Paediatrics, Medical School, University of Pécs, Pécs, Hungary; ^2^ National Laboratory on Human Reproduction, University of Pécs, Pécs, Hungary; ^3^ Doctoral School of Health Sciences, Faculty of Health Sciences, University of Pécs, Pécs, Hungary; ^4^ Department of Obstetrics and Gynaecology, Medical School, University of Pécs, Pécs, Hungary; ^5^ Obstetrics and Gynecology, Magyar Imre Hospital Ajka, Ajka, Hungary

**Keywords:** obesity, metabolic syndrome, maternal, nutrition, epigenetic, DNA methylation

## Abstract

Growing evidence shows that maternal nutrition from preconception until lactation has an important effect on the development of non-communicable diseases in the offspring. Biological responses to environmental stress during pregnancy, including undernutrition or overnutrition of various nutrients, are transmitted in part by DNA methylation. The aim of the present narrative review is to summarize literature data on altered DNA methylation patterns caused by maternal macronutrient or vitamin intake and its association with offspring’s phenotype (obesity and related metabolic changes). With our literature search, we found evidence for the association between alterations in DNA methylation pattern of different genes caused by maternal under- or overnutrition of several nutrients (protein, fructose, fat, vitamin D, methyl-group donor nutrients) during 3 critical periods of programming (preconception, pregnancy, lactation) and the development of obesity or related metabolic changes (glucose, insulin, lipid, leptin, adiponectin levels, blood pressure, non-alcoholic fatty liver disease) in offspring. The review highlights that maternal consumption of several nutrients could individually affect the development of offspring’s obesity and related metabolic changes via alterations in DNA methylation.

## 1 Introduction

The prevalence of obesity has become a global epidemic worldwide, that affects people of all ages. In the European Region, almost 60% of adults and nearly one in three children are obese or overweight according to the report of the World Health Organization (1). Obesity-related non-communicable diseases (metabolic syndrome (MS), type 2 diabetes mellitus, and cardiovascular disease), are linked to increased mortality. Parallel with the increase of obesity, the prevalence of MS is also rising already in childhood and adolescence (2), therefore the investigation of underlying mechanisms behind their development is an emerging field.

Obesity occurs through different mechanisms, and it is an archetype of multifactorial diseases. According to the developmental origins of health and disease (DOHAD) hypothesis, inadequate nutritional exposures during the three critical periods (preconception, pregnancy, and lactation/early postnatal period) may play a critical role in determining the risk of obesity in the progeny (3, 4).

While the original DOHAD studies mainly concentrated on maternal undernutrition (where the consumption of all nutrients was insufficient at the same time), there is also growing evidence demonstrating an association between inadequate maternal intake of individual macro and micro-nutrients during the perinatal period and the development of obesity and related metabolic changes in the progeny (5). Both intrauterine malnutrition and overnutrition of different nutrients may represent a stress state for the embryo and may cause early programming that increases the risk of adult chronic disease development (6). Epigenetic mechanisms in the background of intrauterine programming have been proposed as one of the underlying effects (7). Gene expression is modulated by epigenetic mechanisms without altering the DNA sequences (7). DNA methylation, small non-coding microRNAs, and histone modifications are the main components of epigenetic mechanisms. One of the most studied epigenetic modifications is DNA methylation. DNA is methylated at the cytosine bases at the carbon-5′ position in cytosine-guanine dinucleotide residues. The methyl group addition affects the gene expression by (usually) inhibiting transcription. Alterations in the methylation of promoter regions of several genes are responsible for the plasticity of early programming. Changes in DNA methylation can occur throughout life, but much of the epigenome is established during embryogenesis and early development of the fetus (8). Shortly after fertilization both the paternal and maternal genomes are demethylated followed by *de novo* methylation just before implantation (9). These are critical periods in the growing embryo where nutritional factors may influence the methylome (9). Evidence mostly from animal models shows that changes in early postnatal nutrition quality and/or intake influence offspring’s epigenetic and expression profile in various organs including body weight regulation, suggesting the early postnatal period as another critical window of epigenetic regulations (10). During cell division, many epigenetic changes that occurred throughout the development (pre- and postnatally) survive, resulting in constant consequences of these marks on gene expression throughout life (11) that may predispose individuals to an altered metabolism resulting in the development of non-communicable diseases in later life (12).

In recent times, the number of animal and human studies investigating the epigenetic effect of maternal over- or undernutrition of several macro- and micronutrients on the development of obesity and related metabolic changes in the offspring has increased. In most of the previous investigations, the combined effect of several types of nutrients has been studied, which makes the interpretation difficult. Consequently, it is inevitable to investigate each macro- and micronutrient individually to better understand the exact molecular basis behind it. For some of the investigated nutrients, like nutrients involved in the one-carbon metabolism, the exact role in DNA methylation is clearly described, while for other nutrients (long-chain polyunsaturated fatty acids (LCPUFA), vitamin D, fructose) the clear mechanism of how the nutrients can alter DNA methylation pattern remains an important topic of research.

The purpose of this narrative literature review is to summarize the most recent data concerning alterations of offspring’s DNA methylation patterns caused by maternal macronutrient, vitamin or methyl-group donor intake during pregnancy leading to the development of obesity and related metabolic changes in the offspring.

## 2 Materials and methods

This narrative review specifically focuses on published studies investigating maternal individual nutrient intake before conception, during pregnancy, and lactation + alterations in DNA methylation pattern of offspring + measurements included obesity development and/or related metabolic changes (dyslipidemia, hyperglycemia, insulin resistance, hypertension, fatty liver disease) in offspring in the same study.

The literature search was conducted in PubMed, Medline, and the Cochrane Library in May 2023 using keywords: 1) maternal nutrition AND epigenetic AND protein, 2) maternal nutrition AND epigenetic AND fat, 3) maternal nutrition AND epigenetic AND carbohydrate, 4) maternal nutrition AND epigenetic AND vitamin, 5) maternal nutrition AND epigenetic AND folate.) with restriction of the languages (included articles published in English).Animal and human studies were included where the associations of the three following parameters were investigated together: 1. the DNA methylation pattern of at least one gene of the offspring; 2. data on at least one of the individual nutrient intakes of the mother during pregnancy; 3. at least one of the outcome measures included obesity development or related metabolic changes in the offspring. Those manuscripts not fulfilling the above-described criteria, or investigating *in vitro* models were excluded.

To summarize those investigations where the epigenetic effect of maternal starvation, maternal overnutrition or obesity and diabetes on the development of offspring obesity and related metabolic changes was described without a detailed description of the nutrients involved in the starvation/overnutrition is beyond the scope of this narrative review. The studies where the epigenetic effect of maternal intake of a combination of different nutritional groups was investigated were excluded from this narrative review (except for the methyl-group donor nutrients). A comprehensive review of the exact mechanisms of how individual nutrients affect epigenetic alterations is beyond the scope of this narrative review.

## 3 Results

### 3.1 Association between maternal carbohydrate overnutrition during pregnancy, DNA methylation pattern, obesity and related metabolic changes in the offspring

A current review summarized the role of maternal carbohydrate overnutrition on the early origin of MS in the offspring (13). The authors found an overall effect of the excess maternal carbohydrate intake on increased body weight and hepatic lipid content of the offspring (particularly in males). However, high sucrose supplementation led to favorable metabolic outcomes of female progeny in several rat models (13). The consumption of nutrients containing fructose has increased during pregnancy in recent decades, despite rising evidence about its numerous negative effects on offspring’s health (development of obesity, hepatic steatosis, dyslipidemia, insulin resistance, hypertension, and hypoadiponectinaemia) (14–19).

Results from studies suggest a role of maternal fructose intake (independent of other nutrients) on offspring’s DNA methylation pattern of different genes (20, 21) leading to the development of obesity and related metabolic changes in the offspring (results summarized in **Table 1**). Our literature research was not able to identify similar studies on the independent effect of other monosaccharides or di- and polysaccharides.

**Table 1:** Summary of studies investigating the maternal intake of carbohydrates during pregnancy on the development of offspring’s obesity and related metabolic changes via alteration in the DNA methylation pattern of different genes.

Study author, year	Investigated subject, number of subjects	Mothers and offspring’s diet during the study	Site of samples and observed alteration in DNA methylation	Effect on obesity development and related metabolic changes
Animals studies				
Rodrigo 2018 (15)	Sprague–Dawley rats n=18 (6/group)	Mothers diet: -during gestation: fructose or glucose in drinking water (10% w/v solution) and control group (no supplementary sugar) -after delivery: maintained with food and water ad libitum Offspring’s diet: -suckling was stopped on the 21 st day -maintained with food and water ad libitum	Liver tissue, PD: 261. Fructose-supplemented group males: -LXRα gene promoter methylation: ↓ females: -LXRα gene promoter methylation: ↑	PD 261: in the fructose-supplemented group (vs. glucose-supplemented group) -in males: plasma HDL-cholesterol levels ↑ -in females: lower levels of non-HDL cholesterol
Yamazaki 2020 (22)	Sprague–Dawley rats Fructose group n=19 Control group n=21	Mothers diet: during gestation and lactation (until PD 21): -fructose supplemented group: 20% fructose w/v solution -control group: distilled water Offspring’s diet: -standard chow diet and distilled water from weaning to PD 160	Liver tissue, PD 21, PD 60 and PD 160. DNA methylation in the promoter region of LXRα showed no difference	PD 70 to PD 160: In the fructose-supplemented group, the body weight was significantly ↓ vs. the control group. At any time point: caloric intake was NS different between groups PD21, PD60, and PD160: -NS difference in the serum concentrations of TG, T-cho, and non-HDL-cho between groups
Yoshitaka Ando, 2022 (16)	Sprague–Dawley rats n=26	Mothers diet: during gestation and lactation (until PD 21) -fructose supplemented group (HFCS): 20% high-fructose corn syrup (HFCS) water solution + standard chow -control group (C): distilled water + standard chow Offspring’s diet: standard chow and distilled water ad libitum until PD 60	Liver tissue: PD 21: -NS in DNA methylation of *Cpt1a* between groups PD 60: -NS in DNA methylation of *Cpt1a* between groups - DNA methylation ↑ in the promoter region of Pparα in HFCS compared to C	PD 21 and PD 60: -NS changes in the body weight and caloric intake in the offspring of both groups PD 21: -NS differences in serum TG, Glu, T-Cho, and leptin levels between groups -serum adiponectin significantly ↑ in the HFCS compared to C PD 60: -NS differences in serum Glu, T-Cho, and leptin levels -serum TG significantly ↑ in the HFCS compared to C -NS differences in hepatic LPO and TG levels -during ivGTT: NS differences in glucose AUC between groups, but insulin AUC level was ↑ in the HFCS group

PD, postnatal day; NS, no significant; TG, triglyceride; T-cho, total cholesterol; non-HDL cho, non-HDL-cholesterol; Glu, glucose; LPO, lipid hydroperoxide; ivGTT, intravenous glucose tolerance test; AUC, area under the curve; Pparα, Peroxisome proliferator-activated receptor alpha; LXRα, Liver X receptor alpha.

↓, decreased; ↑, increased.

In a study, where pregnant rats were fed with a 20% fructose-containing water solution during gestation and lactation, the progeny developed hyperlipidemia and insulin resistance at 60 days of age (only male offspring were investigated in this study) (16). Furthermore, hepatic peroxisome proliferator-activated receptor α (Pparα) expression was suppressed by the increased DNA methylation of the promoter region compared to controls suggesting a role of Pparα hypermethylation caused by excessive fructose intake in the development of hyperlipidemia and insulin resistance in the progeny (16).

In another study, 10% w/v solution fructose or glucose was supplied to pregnant rats throughout gestation. Elevated plasma HDL-cholesterol levels were observed in the male progeny and lower levels of non-HDL cholesterol were presented in the female progeny from fructose-supplemented mothers compared to glucose-supplemented or control groups. Genes (Liver X-receptor, scavenger receptor B1, ATP-binding cassette (ABC)G5 and cholesterol 7-alpha hydroxylase gene) playing an important role in cholesterol metabolism showed decreased gene expression in males and increased in the female offspring from fructose-supplemented mothers. Furthermore, Liver X-receptorα gene (LXRα) promoter methylation elevated in males and decreased in the corresponding group of females from mothers fed fructose (15).

Yamazaki et al. investigated the epigenetic effect of maternal fructose supplementation (20% fructose w/v solution) on the lipid status of male rat offspring and found decreased serum HDL-cholesterol levels and decreased LXRα gene expression in the offspring’s liver tissue on the 160th postnatal day, however, in this study no differences in DNA methylation level of LXRα promoter region could be detected (22).

These studies in animals suggest that a high fructose diet during pregnancy is associated with decreased HDL-cholesterol levels (that may be gender-specific for females), hyperlipidemia, and insulin resistance through alterations in the methylation pattern of certain genes (LXRα and Pparα) playing an important role in the regulation of lipid homeostasis, the oxidation and transport of fatty acids. The above studies were conducted in animals, but in fact, the 10% w/v of fructose supplementation is similar to sugary beverages and the amount of calories originating from simple sugars (glucose, fructose) in the above investigations is very close to the energy intake of heavy consumers of sugary beverages in humans (23). Consequently, these studies may suggest an epigenetic role of maternal consumption of fructose containing beverages in the development of obesity and related metabolic changes in the offspring of humans as well.

### 3.2 Association between maternal protein intake during pregnancy, DNA methylation pattern, obesity, and related metabolic changes in the offspring

Animal studies show growing evidence that the progeny of mothers with low protein diet during pregnancy become more obese, hypertensive, and glucose intolerant as compared to the offspring of dams consuming standard diet (24–26). The offspring of mothers exposed to high protein consumption during pregnancy have higher body fat content and levels of signaling molecules associated with reduced insulin sensitivity as they age compared to progeny of control dams (the mother consuming a standard diet) (27). The results in humans are more conflicting, as inverse, direct, and null associations were also published between maternal intake of protein and obesity development in the offspring (28). Epigenetic mechanisms may stand behind these associations, as some amino acids (e.g., methionine, serine, glycine, and histidine) play an important role in the supply of methyl donors for the methylation of the DNA (29). Branched-chain amino acids stimulate insulin secretion, which may lead to insulin resistance and obesity (30, 31). Epigenetic studies provide evidence that inappropriate maternal protein intake can lead to alteration in DNA methylation in genes having a role in the regulation of growth, body composition, and metabolism (lipid, carbohydrate homeostasis, and blood pressure regulation) in the progeny (32–36), however, the number of investigations studying the effect of the alterations in DNA methylation induced by maternal protein malnutrition on the development of obesity or related metabolic changes in the offspring is very limited (**Table 2**). In a study, pregnant sows were divided into three diet groups during pregnancy: control group (CO), containing 12% crude protein (CP), low protein group (LP): containing 6% CP, and, high protein group (HP): containing 30% CP. The body weight of fetuses did not differ between CO, HP, and LP groups at gestational day 95. Significantly lower birth weight was observed in LP in comparison to CO pigs. At postnatal days 28 and 185 no differences in body weight were observed among CO, HP, and LP groups. Protein restriction had a significant effect on hepatic global methylation at gestational day 95 (38).

**Table 2:** Summary of studies investigating the maternal intake of protein during pregnancy on the development of offspring obesity and related metabolic changes via alteration in the DNA methylation pattern of different genes.

Study author, year	Investigated subject, number of subjects	Mothers and offspring’s diet during the study	Site of samples and observed alteration in DNA methylation	Effect on obesity development and related metabolic changes
Animals studies				
Jousse 2011 (37)	Balb/c mice n=20	Mothers diet: during gestation and lactation: - low-protein diet (LPD) (10% protein) -control group (CD: containing 22% protein) Offspring’s diet: after weaning (4 wk) the male offspring were given a standard chow diet	Adipose tissue, age: 7 months. The promoter of the leptin gene was hypomethylated	From PD 10 onwards (throughout life): significantly ↓ body weight of LPD than CD male mice. At the age of 7 months: -significantly ↓ lean and fat masses, percentage of fat in LPD than in CD mice. -In the LPD group, daily food consumption relative to individual mouse body weight was significantly ↑
Altmann 2012 (38)	German Landrace sows n=16/diet group	Mothers diet: during gestation: -control group (CD): 12% crude protein (CP) -high protein group (HPD): 30% CP; -low protein group (LPD): 6% CP Offspring’s diet: after weaning (PD 28) offspring had ad libitum access to standard commercial diets	1) Liver: At gestational day 95: -global DNA methylation was significantly ↓ in LPD relative to CD offspring At PD 1, and 28: -NS difference among groups in global DNA methylation At PD 188: -significantly ↓ global methylation in HPD than CO 2) Skeletal muscle. At gestational day 95 and at PD 1, 28, 188: NS difference in global methylation among groups .	At gestational day 95: -Weight did not differ among CD, HPD, and LPD groups. LPD piglets had significantly lower birth weight compared to CD. At PD 28 and 128: -No differences were found in body weight among CD, HPD, and LPD groups.

NS, no significant; PD, postnatal day; LPD, low protein diet; CD, control diet group; HPD, high protein group.

↓, decreased; ↑, increased.

Higher food intake and lower body weight were observed in the mice offspring of mothers consuming a low-protein diet (LPD) (10% protein) compared to mice born to mothers consuming a control diet. Moreover, the removal of methyl-groups from the promoter region of the leptin gene was observed by the offspring (37).

The most investigated amino acid in relation to the development of epigenetically induced obesity is methionine, which plays a significant role in the one-carbon metabolism as a methyl-group donor for DNA methylation. The literature search could not identify publications where the association between methionine intake of the mother during pregnancy influenced the DNA methylation pattern of the offspring and as a consequence led to the development of obesity and related metabolic changes.

These animal studies suggest that a low-protein diet during pregnancy is associated with lower birth weight and the development of obesity and lower blood glucose concentration through alterations in the methylation pattern of certain genes (LEP, G6PC, and alteration in global hepatic DNA methylation) having an important role in the regulation of food intake, and glucose homeostasis (37, 38).

### 3.3 Association between maternal high-fat diet and LCPUFA intake during pregnancy, DNA methylation pattern, obesity, and related metabolic changes in the offspring

The development and growth of the offspring are affected by the dietary fat intake of the mother during pregnancy and lactation. The International Society for the Study of Fatty Acids and Lipids (ISSFAL) and the consensus statement for dietary fats have stated that the dietary fat consumption throughout gestation, as a proportion of energy intake, should not be different as that is recommended for the general population (39). Recent animal and human studies show that a maternal high-fat diet (HFD) has an effect on the general health of the offspring including the development of obesity, diabetes mellitus, and cardiovascular diseases (40–43). Some of these phenotypic differences caused by a maternal HFD have been associated with epigenetic changes through several pathways (44).

#### 3.3.1 High-fat diet

In a study by Marco et al. the progeny of mother rats fed HFD (60% fat) during gestation had higher birth weights and ate more postnatally, leading to higher body weight at the age of 110 days compared to controls (45). Hypermethylation across the promoter region of the Pro-opiomelanocortin (POMC) was observed in the offspring from the HFD group (only female offspring were investigated).

In another study, researchers found elevated blood pressure (at 6 months and at 1 year), and increased levels of leptin (at 3 and 8 weeks, 6 months, and 1 year) in the offspring of pregnant rats exposed to HFD (HFD: 18.7% lard, 2.5% maltodextrin, 11.4% sucrose, 12% casein, 0.3% cholate, 1.3% cholesterol, and 52% standard chow) during pregnancy compared to chow-exposed offspring (the ratio of males to females offspring was 1:1 in every group). The HFD-exposed pups had a higher birth weight and higher body weight at 3 weeks of age compared to controls, but this observation no longer existed at 8 weeks after birth. In addition, an increased expression of the leptin gene and hypomethylation of the leptin promoter was found in the adipose tissues of the HFD-exposed progeny (46). In the same study, the authors found elevated blood pressure, serum leptin levels and significantly reduced overall DNA methylation of the leptin promoter in preschool-aged children born to women with hypertriglyceridemia (serum triglyceride > 3.28 mM) during gestation (46).

In a maternal HFD mouse model (containing 60 kcal% fat), decreased DNA methylation at the promoter of the leptin gene and hypermethylation at the promoter region of the leptin receptor and adiponectin genes with higher plasma levels of leptin and lower levels of adiponectin were reported in the adult offspring (results are presented with males and females merged). In contrast to the study of Lin et al. in this study, the authors could not observe differences in birthweight in the HFD pups compared to the low-fat diet controls, but in the post-natal period (week 6 of age), HFD offspring had higher body weight and consumed more calories compared to controls. Furthermore, HFD pups exhibited insulin resistance and hyperlipidemia (47).

Male offspring of maternal HF (60% fat) diet had rapid early weight gain, increased adiposity, and hyperleptinemia compared to offspring of low fat (10% fat) diet at 2 days and 3 weeks of life. The *Pomc* gene from the arcuate nucleus punches showed hypermethylation in the enhancer (nPE1 and nPE2) regions and in the promoter sequence mediating leptin effects (48).

In mice, maternal HFD (45% fat) had no significant effect on progeny’s body weight but was associated with hyperglycemia and glucose intolerance in male pups. The livers of offsprings exposed to a maternal HFD had hypomethylation in the mitogen-activated protein kinase 4 (Map2k4) gene and hypermethylation in the insulin receptor substrate 2 (Irs2) gene, which are involved in the insulin signaling pathway and the MAPK pathway (49). No difference in birth weight was observed in the progeny of mothers consuming HFD (45% fat) compared to controls, but HFD was associated with the development of non-alcoholic steatohepatitis and hypermethylation in genes implicated in lipid accumulation and liver fibrosis (Both male and female offspring were studied separately. However, since phenotypes of liver pathology were most prominent in males, only data from male offsprings were presented.) (50).

These studies in animals and humans suggest that HFD in pregnancy is associated with the development of offspring’s obesity and related metabolic changes through alterations in the methylation pattern of several genes (POMC, LEP, Map2k4, Irs2, Ephb2, Fgf21) having an important role in the regulation of energy balance, food intake, insulin resistance, and hypertension. However, drawing conclusions from these studies is difficult, as the time period of HFD of mothers varies (before pregnancy, during pregnancy, lactation period). A summary of the studies is presented in **Table 3**.

**Table 3:** Summary of studies investigating the maternal intake of fats during pregnancy on the development of offspring’s obesity and related metabolic changes via alteration in the DNA methylation pattern of different genes.

Study author, year	Investigated subject, number of subjects	Mothers and offspring’s diet during the study	Site of samples and observed alteration in DNA methylation	Effect on obesity development and related metabolic changes
Animals studies				
Khalyfa, 2013 (47)	wild-type C57BL/6J mice n=36	Mothers diet: Following 12 days of pregnancy and during the lactation (until PD 21): - HFD (60 kcal% fat), - LFD (10 kcal% fat) Offspring’s diet: after weaning (PD 21) access to water and were fed with LFD for 18 weeks, and were killed at age 21 weeks	Liver tissue, visceral fat and skeletal muscle, at 21 weeks of age. DNA methylation at the promoter of the leptin gene ↓ in visceral fat, skeletal muscle and liver tissue in HFD group Methylation at the promoter region of the leptin receptor ↑ in the visceral fat and liver tissue Methylation of the adiponectin genes ↑ in the visceral fat and skeletal muscle tissue Methylation of the GLP-1 showed no significant changes in visceral fat, liver or skeletal muscle between groups	-No difference in birth weight among groups From postnatal weeks 6 until 21 weeks: In the HFD group: body weight ↑, food intake↑, At 20 weeks of age: -significant ↑ fat mass in subcutaneous and visceral adipose tissues in the HFD group -the HFD offspring exhibited insulin resistance, hyperlipidemia (↑ fasting T-cho, ↑ LDL-cho, ↑ TG, ↑ serum-free fatty acid, and ↓ HDL-cho), ↑ plasma levels of leptin, ↓ levels of adiponectin when compared with LF
Marco, 2014 (45)	Wistar rats n=40	Mothers diet: from PD 22 to 80 days (beginning of pregnancy) -HFD (60% fat) -Control diet (6% fat) Offspring’s diet: after weaning (PD 22) control diet	Brain tissue, PD 22: In the HFD group: hypermethylation across the promoter region of the Pro-opiomelanocortin (n=22)	-↑ birth weight in the HFD group From PD 22 throughout life: -↑ body weight in HFD group -HFD offspring consume ↑ kilocalories per day,
Wankhade, 2017 (50)	C57BL6/J mice n=20	Mothers diet: From 5 weeks old until beginning of pregnancy (17 weeks of age) -HFD (45% fat) -Control diet (17% fat) Offspring’s diet: after weaning: access to control or HFD diet. 1)Control diet dam, control diet offspring (CC) n = 10) 2)Control diet dam, HFD diet offspring (CH, n = 11) 3) HFD dam, C diet offspring (HC, n = 7) 4) HFD diet dam, HFD diet offspring (HH, n = 7)	Liver tissue. DNA methylation analysis of over 1 million CpGs showed an association between epigenetic alterations in genes playing a role in the metabolism and development of the tissues (Fgf21, Ppargc1β), cell adhesion and communication (VWF, Ephb2) and maternal HFD	In the HFD group: -no difference in birth weight PD 126: - ↑ body fat mass in HH vs, CH, -serum glucose and triglycerides were not different between the groups -cholesterol and NEFA were significantly affected by maternal HFD and combination of maternal and offspring HFD, -in HH offspring relative liver weight ↑, -in CH and HH offspring hepatic steatosis,
Ramamoorthy, 2018 (48)	Sprague–Dawley rats n=20	Mothers diet: 6 weeks before conception, throughout gestation, during lactation (PD 21) -HFD (60% fat) -Low-fat diet (LFD) (10% fat) Offspring’s diet: -Until PD 21 weaned by the mother	Arcuate nucleus, age: 3 weeks The *Pomc* gene showed hypermethylation in the enhancer (nPE1 and nPE2) regions and in the promoter sequence mediating leptin effects	At PD 2 and PD 21: In the offspring of HFD group: ↑ body weight (↑ in both fat mass [epididymal, mesenteric, as well as subcutaneous fat weigh] and non-fat mass) -↑ plasma leptin level at 3 weeks of age in the offspring of HFD
Zhang, 2019 (49)	C57BL6/J mice n=40	Mothers diet: 4 weeks before pregnancy + during pregnancy + during lactation: -HFD: (20, 35, and 45% of calories from protein, carbohydrate and fat) -Control diet (CFD) group: (20, 64, and 16% of calories from protein, carbohydrate, and fat) Offspring’s diet: -Until PD 21 weaned by the mother -From PD 21 until 8 weeks of age: 1)pups from CFD dams were weaned onto the control diet (CFD-CFD) or HFD diet (CFD-HFD). 2)pups from HFD-fed dams were weaned onto the CFD (HFD-CF) or HFD (HFD-HFD).	Liver tissue, age: 8 weeks. -a total of 1,099 differentially methylated regions were identified on 20 chromosomes in the HFD-CFD group compared with the CFD-CFD group -Hypomethylation in the mitogen-activated protein kinase 4 (Map2k4) gene -Hypermethylation in the insulin receptor substrate 2 (Irs2) gene	At 8 weeks of age: -the mean body weight of mice in the CFD-HFD and HFD-HFD groups was significantly ↑ than that of CFD-CFD and HFD-CFD mice -NS maternal HFD effect on offspring body weight -Association with hyperglycemia (Offspring from HFD-fed dams displayed ↑ fasting blood glucose) and glucose intolerance in male pups
Lin et al., 2021 (46)	Sprague–Dawley rats n=40	Mothers diet: pregnant females were fed chow or an HFD from 8 weeks to delivery: - HFD (11.4% sucrose, 18.7% lard, 12% casein, 1,3% cholesterol, 0.3% cholate and, 52% maltodextrin) -Control diet group (CFD) Offspring’s diet: After weaning, the HFD-exposed offspring were given the same diet as the controls	Adipose tissue and lymphocytes, age: 3 weeks old. -overall DNA methylation levels of the *leptin* gene were significantly reduced in the fetal adipose tissue, subcutaneous-, and visceral adipose tissue of HFD offspring	At birth: - ↑ body weight in HFD group In the HFD group: -systolic blood pressure ↑ (at 6 months and 1 year old) -levels of leptin ↑ (at 3 and 8 weeks, 6 months, and 1 year) -↑ body weight at 3 weeks of age -↑ fat deposit in males at 3 weeks old At 8 weeks after birth, the differences in the body weight and in the percentage of adiposity were not anymore detectable between the control and the HFD pups.
Human studies				
Lin et al., 2021 (46)	Humans n=121	Third-trimester serum triglyceride levels were analyzed. 1) Women with gestational hypertriglyceridemia (serum triglyceride > 3.28 mM) 2) Women with low serum triglyceride ≤ 3.28 mM) groups	Lymphocytes, age: newborn and preschool-age (3-6 years old). Significantly reduced overall DNA methylation of the leptin promoter in children born to high TG level mothers	In children of women who had hypertriglyceridemia during pregnancy: -higher birth weight -blood pressure ↑ in male preschool-age children -levels of leptin ↑ in male preschool-age children

HFD, high-fat diet; PD, postnatal day; NS, no significant; TG, triglyceride; T-cho, total cholesterol; LDL-cho, Low-Density Lipoprotein-cholesterol; NEFA, Non-esterified fatty acids; Fgf21, Fibroblast growth factor 21; Ppargc1β, Peroxisome proliferator-activated receptor gamma coactivator 1-beta; VWF, von Willebrand factor; Ephb2, Ephrin type-B receptor 2; Map2k4, mitogen-activated protein kinase 4.

↓, decreased; ↑, increased.

#### 3.3.2 LCPUFA

Long-chain polyunsaturated fatty acids (LCPUFAs) including docosahexaenoic acid (DHA) and eicosapentaenoic acid (EPA) are essential in the development of the fetus. LCPUFAs on the n-3 and n-6 series are obtained by nutrition or produced by polyunsaturated fatty acid (PUFA) precursors. Since the DHA synthesis is reduced in the developing fetus, it must be obtained through the placenta from the mother (51). The ISSFAL and the consensus statement for dietary fats developed a recommendation for pregnant women only for the DHA intake: during pregnancy, it is recommended to consume at least 200 mg/day of DHA, which can be obtained by consuming one to two portions of sea fish (including oily fish) per week (39).

Previous studies of maternal supplementation with some LCPUFAs (Docosahexaenoic Acid, Arachidonic Acid, and Eicosapentaenoic Acid) have shown an association between a higher duration of pregnancy, reduced risk of preterm delivery, higher birth weight, and between brain development and visual function of the offspring (39, 52, 53). Until now, only a few studies have investigated the potential benefits of maternal n-3 LCPUFA intake in preventing offspring’s risk of obesity and related metabolic changes and the results are still conflicting (53). Many factors, including epigenetic mechanisms, may contribute to the observed effects. In recent times, some studies conducted in large, placebo-controlled, randomized trials reported an association between the maternal consumption of DHA and alteration in the offspring’s DNA methylation pattern in different genes, playing a significant role in appetite regulation or in the development of low birth weight, hypertension, diabetes, and cardiovascular diseases (54–56). However, the presence of obesity and related metabolic changes in the offspring was not investigated in the same study.

### 3.4 Association between maternal vitamin intake during pregnancy, DNA methylation pattern, obesity, and related metabolic changes in the offspring

Animal studies suggest that a higher multivitamin consumption during gestation increases body weight, food intake, and characteristics of the MS of the progeny (57–59). The number of studies investigating the role of altered DNA methylation patterns caused by maternal vitamin intake (especially the vitamins playing a role in the one-carbon metabolism and vitamin D) during pregnancy in the development of obesity and related metabolic changes has increased recently (58, 60) (**Table 4**).

**Table 4:** Summary of studies investigating the maternal intake of vitamins and methyl-group donors during pregnancy on the development of offspring’s obesity and related metabolic changes via alteration in the DNA methylation pattern of different genes.

Study author, year	Investigated subject, number of subjects	Mothers and offspring’s diet during the study	Site of samples and observed alteration in DNA methylation	Effect on obesity development and related metabolic changes
Animal studies				
Sinclair 2007 (61)	Scottish Blackface ewes n=310	Mothers diet: 8 weeks before until 6 days after conception: -methyl-deficient diet (MD): reduced amount of folate, B12 and methionine -control group Offspring’s diet: All offspring were offered a standard diet from weaning, which was balanced for all major and micronutrients, with supplemental hay	Liver tissue, gestational day 90. 57 loci were altered from the 1,400 CpG loci investigated in at least 2 MD ewes. 88% of the loci were hypomethylated or unmethylated compared to the control group.	At 12 months of age: no differences in body fatness between treatment group At 22 months of age: -Female body composition was unaffected by experimental treatment. -MD males were proportionately fatter than control males and had less muscle mass -MD male offspring had increased resistance to insulin, At 23 months of age: - relative to their respective controls, systolic, diastolic, and mean arterial pressor responses were ↑ in MD male, but not in MD female offspring -adult male progeny were more obese and had altered immune function
Giudicelli 2013 (62)	Sprague Dawley rats n=32 (n=8/group)	Mothers diet: for 21 to 28 days before mating, and throughout gestation and lactation (PD 21): (a) control diet (C), (b) C + MD diet, (c) protein restricted diet (PR) (d) PR + MD methyl-donor (MD) diet: folic acid, methionine, choline, betaine, Vitamin B12 and Zn Offspring’s diet: **-**weaned until PD 21 -from PD 21 until the age of 42 days: the standard growth diet -fed a standard maintenance diet until the age of 23 weeks. -At 23 weeks, all animals were given free access to a hypercaloric high-fat high-sucrose western diet for 4 weeks	White adipose tissue, age: PD 21. Hypermethylation of LEP promoter	- Slightly heavier birthweight in PR compared to C group - Lower birth weight in C+MD compared to C group At the age of 21 days: -Postnatal growth was impaired in PR and PR+MD compared to C -Plasma glucose ↓ in PR+MD and ↑ in C+MD and PR and PR+MD compared to C group -Leptin concentration ↓ in C+MD and PR+MD compared to C group
Sanchez-Hernandez 2015 (58)	Wistar rats n=14/16 per group	Mothers diet: during pregnancy: -high-fat soluble vitamin diet (HFS; 10-fold vitamins D, A, K, and E) -multivitamin diet (1-fold all vitamins) During lactation, all dams were fed the control diet. Offspring’s diet: were maintained on the control diet for 14 weeks	Hippocampus tissue. -At any time period (at birth, weaning, and 14 weeks postweaning): Gestational diet did not influence global DNA methylation At birth: -Diet had no effect on promoter regions of Dat and Drd5 genes -Postweaning: DNA hypermethylation in Drd1 promoter region in HFS group was detected, diet had no effect on Drd5 methylation	At 2 weeks postweaning: -preference for sucrose ↓ by 4% in the offspring of dams consumed the HFS diet -Oil preference was not changed From week 0 to week 12 postweaning: Food intake and body weight were not different between groups
Xue 2016 (63)	Mice CC001/Unc (CC001) and CC011/Unc (CC011) mice FVB/NJ (FVB) mice (unexposed)	Mothers diet: For CC001 and CC011 G_0_ mice, 5 weeks before mating and throughout gestation and weaning (PD 21): -Control diet (1000 IU/kg of vitamin D_3_) -Diet not containing vitamin D (LVD) Offspring’s diet: offspring were transferred at weaning (PD 21) to standard rodent chow First generation male offspring (cross 1: G_1=_ generated from CC001 females × CC011 male; cross 2: G1 = generated from CC011 females x CC001 males) G_1_males + FVB females: G_2_ litters	Liver tissue, sperm samples, age: 8 weeks. -In G_1_: Significantly lower methylation was observed in LVD liver compared with controls at the *H19PP*, *H19/Igf2* ICR and *Grb10DMR* -no diet dependent methylation differences were observed in sperm	- LVD was not associated with fecundity, fertility, or progeny’s postnatal viability At 8 weeks of age: -significantly ↑ testes and body weight was observed in Cross 1 G_1_ LVD males compared to controls -cross 2 G_1_ LVD males did not differ in testes weight but exhibited a slight trend in ↓ body weight compared to controls - In the G_2_ offspring at PD 4: cross 1 G_2_ LVD males exhibited significantly ↑ body weights while cross 2 LVD males exhibited significantly ↓ body weights compared with controls By adulthood: -diet-dependent G_2_ male body weight differences were no longer significant for either cross individually or combined - In the males of cross 1 G_1_ a negative correlation between DNA methylation at Grb10 DMR and testis and body weight was observed at the age of 8 weeks old
Meems 2016 (64)	Sprague-Dawley Rats n=14 (7/8 per group)	Mothers diet: 10 weeks before pregnancy + during pregnancy: -normal chow (C) (n=14) -D vit depleted diet (n=16) (VDD) After delivery: standard diet that included vitamin D Offspring’s diet: weaned at 3 weeks onto a standard chow diet	Heart or kidney tissue, age: 10-12 weeks. In the kidney tissue of F1-VDD rats a significant hypermethylation of the Panx1 was detected vs. F1-C rats	At 6 weeks of age: F1-VDD offspring had significantly ↑ systolic and diastolic blood pressure compared to F1-C offspring At the age of 10-12 weeks: -No differences in total heart weight, left ventricular weight, and body weight between F1-VDD and F1-C -Significant difference in acetylcholine-induced relaxation of F1-VDD offspring in contrast to F1-C offspring -↑ atrial natriuretic peptide was observed in F1-VDD offspring compared to F1-C offspring
Wen 2018 (65)	Sprague-Dawley rats n=43	Mothers diet: from 4 weeks of age + during gestation: -Control group: vitamin D replete diet (1000 I.U/kg) (C) -Vitamin D deficient diet (0 I.U/kg) (VDD) From birth, all rats consumed vitamin D replete diet. Offspring’s diet: vitamin D replete diet.	Adipose tissue, age: 14 weeks. In the VDD group, 204 CpG islands and 608 promoters and were differentially methylated, involving 305 genes	No significant difference in birthweight between C and VDD From the age of 10 weeks until 14 weeks: ↑ weight in VDD compared to C At the age of 14 weeks: -plasma concentrations of TG, T-chol, LDL-cho, and HDL-cho were significantly ↑ in VDD offspring when compared with C offspring -No association between VDD and alterations of feeding behavior and locomotor activity -Carbon dioxide production, 24 h oxygen consumption, respiratory exchange rate, and heat production were significantly ↑ in VDD compared to C progenyIn the VDD progeny the blood glucose levels were significantly ↑ than that of C progeny at 60 minutes after glucose injection at the age of 6 weeks, and at 15 and 30 min after glucose injection at the age of 14 weeks
Skjærven, 2018 (66)	Zebrafish (AB strain) n=20	Maternal diet: from 27 days post-fertilization until mating or sampling: -diet marginally below or above the requirement levels given for carp, in 1-carbon nutrients (methionine, choline, folate, vitamin B6 and vitamin B12)	Hepatic tissue, at 142 days post-fertilization. The gene expression of lipogenesis and the hepatic methylation of DNA was affected: a decrease in methylation at transcriptional start sites was observed	At 7, 44 and 113 days post fertilization: Parental feed did not affect the body weight in the offspring At 142 days post fertilization: significant ↑ in liver lipid content in offspring from the low 1-carbon nutrient group
Human studies				
McCullough 2016 (67)	Human mother-infant pairs n=496	Blood sampling from mothers was conducted during the first trimester, vitamins B_12_, B_6_, and homocysteine concentrations were determined	Cord blood. -Methylation at the MEG3 DMR was positively associated with maternal pyridoxal phosphate concentrations -Maternal micronutrient levels did not affect DNA methylation at other DMRs: H19, SGCE/PEG10, and PLAGL1	-The children of mothers in the highest quartile of B_12_ had lower early-life weight gain compared to the children of mothers in the lowest quartile at 3 years of age, -Higher early-life weight gain was detected among male progeny of the mothers in the highest quartile of maternal B6 at 3 years of age.
Neelon 2018 (68)	Humans: Newborn Epigenetics Study (Durham, North Carolina) n=476	Blood sample was collected during pregnancy (no gestational age criterion applied)	Umbilical cord blood. No associations between maternal 25-hydroxyvitamin D (25(OH)D) and methylation status in IGF2, H19, MEST, MEG3-IG, MEG3, PEG3, NNAT, SGCE/PEG10 and PLAGL1, genes	-An association between lower birth weight and the first and second quartiles of 25(OH)D, compared to the fourth as a reference, was observed -↑ 1-year weight-for-length z-scores and higher 3-year BMI z-scores was associated with Q1 of 25(OH)D (compared to Q4)

MD, methyl-deficient diet; VDD, vitamin D deficient diet; TG, triglyceride; T-cho, total cholesterol; LDL-cho, Low-Density Lipoprotein-cholesterol; HDL-cho, High density lipoprotein; Drd1, Dopamine receptor D_1_; Igf2, Insulin-like growth factor 2; Grb10, Growth factor receptor-bound protein 10; MEG3, Maternally Expressed 3; PLAGL1, Zinc finger protein PLAGL1; MEST, Mesoderm-specific transcript homolog protein; NNAT, Neuronatin; PEG3, Paternally-expressed gene 3 protein; SGCE, Sarcoglycan Epsilon; PEG10, Retrotransposon-derived protein PEG10; DMR, Differentially methylated regions.

↓, decreased; ↑, increased.

#### 3.4.1 Multivitamin diet

Throughout gestation, Wistar rats consumed a high-fat soluble vitamin diet (HFS; 10-fold of D, A, K, and E) or a recommended multivitamin diet. In the offspring of rats fed HFS diet during pregnancy the sucrose preference decreased by 4%, but food intake and body weight were not affected. Gene expressions of the hypothalamic orexigenic neuropeptide Y (Npy) and anorexigenic POMC were increased in weaning and adult subjects. However, the gestational diet did not affect the global DNA methylation, a hypermethylation was detected in the promoter region of dopamine receptor 1 (Drd1) (only male offspring were investigated in this study) (58).

#### 3.4.2 Vitamin D

Vitamin D plays an important role in different metabolic processes in the body. Few natural foods contain vitamin D (fatty fish species, fish liver oils, beef, and egg) (69). The skin is the major source of vitamin D in humans, where it is synthesized from its precursor (70). Studies showed relationships between maternal vitamin D status and glucose tolerance, preeclampsia, cesarean section, altered placental vascular pathology, reproductive failure, infection rates, adverse birth outcomes, abnormal fetal growth patterns, and the risk of preterm birth (71, 72). Furthermore, the impact of intrauterine vitamin D deficiency on later health outcomes like bone development, whole-body mineral content, long-term metabolic health - early postnatal overweight, differences in body fat mass, obesity, increased risk for insulin resistance and type 1 diabetes, hypertension -, and immunologically mediated diseases were also shown in several studies (71, 73, 74). The results in these fields are not consistent and are still under debate. Despite the increasing research data showing these associations between the health and development of the progeny *in utero* and in later life and the vitamin D status of the mother, the D vitamin consumption requirement of the mother during pregnancy, is still contradictory (71). Animal and human research show that vitamin D status of the mother throughout gestation may determine the progeny’s health via epigenetic modifications, since vitamin D may alter the expression of various genes, including the genes that indirectly or directly influence DNA methylation. Although, the mechanism behind this remains uncertain and is still under investigation (75).

Vitamin D deficiency of the mother in mice resulted in altered DNA methylation and body weight in two generations of the male progeny (63). Assayed loci in sperm and liver were mainly hypomethylated (63). A multigenerational diet-dependent effect was shown on body composition and body weight, although an effect on testis weight was detected only in the first generation.

In the offspring (10 male and 10 female) of vitamin D-depleted rats, increased systolic and diastolic blood pressure and the hypermethylation of the promoter region of the Pannexin-1 gene were observed (64).

An epigenome-wide DNA methylation study showed that the male progeny of vitamin D-deficient rats had altered methylation at 812 loci linked to 305 genes in the adipose tissue (65). 141 genes were differentially expressed of the methylated genes including 7 genes related to obesity or lipid metabolisms (Spon1, H19, Hif1a, Adrb2, Smad3, Zfp36, Vldlr). These transcriptional and epigenetic alterations were associated with obesity- and related metabolic changes in the progeny: from 10 weeks of age onwards vitamin D-deficient progeny had higher weight compared to controls. When compared with control offspring, total cholesterol, triglycerides, high-density lipoprotein, low-density lipoprotein, adipose tissue volume, and peak blood glucose were significantly increased but did not result in changes in locomotor activity (vertical, fine, and horizontal motor activity) and feeding behavior (food or water intake) in vitamin D-deficient offspring at the age of 14 weeks (in this study only male offspring were investigated) (65).

The Newborn Epigenetics Study (human) found an association between reduced plasma 25-hydroxyvitamin D of the mother and lower birth weight, higher weight-for-length at 1 year, and body mass index (BMI) z-scores at 3 years in offspring (female/male infants=51%/47%). But no association between midterm 25-hydroxyvitamin D levels and DNA methylation (in cord blood samples) of genes playing a role in fetal growth (MEST, IGF2, H19, NNAT, PEG3, SGCE/PEG10, PLAGL1, MEG3 and, MEG3-IG) was detected (68).

This current animal and human studies raise the possibility that vitamin D deficiency throughout gestation may play a significant effect in the development of obesity and related metabolic changes (dyslipidemia, hyperglycemia, and elevated blood pressure) in the offspring, which could be associated with changes in the methylation of different genes (H19/Igf2, Grb10, and Panx1) playing role in body weight and growth regulation and cell proliferation (63–65, 68).

#### 3.4.3 Methyl-group donors playing role in one-carbon cycle metabolism

Growing evidence suggests that maternal intake of vitamins playing a role in the one-carbon metabolism (folate, folic acid, Vitamin B12, and Vitamin B6) can program obesity and the development of non-communicable chronic diseases in offspring (76). This may be mediated by epigenetic processes. For DNA methylation, one-carbon cycle metabolism nutrients are essential (76). As a methyl-group donor, folate is involved in the one-carbon cycle metabolism (76), it is converted to S-adenosylmethionine (SAM). For the methylation of the DNA, SAM will donate a methyl-group (77). Vitamin B12 and B6 are acting as co-enzymes for a source of methyl groups (76).

The deficiency of folate has been associated with numerous birth outcomes such as spontaneous abortion, preterm birth, low birth weight, and neural tube defects (78, 79). Humans cannot synthesize folate, they have to obtain it from dietary sources (beans, cereals, liver, egg yolks, and green leafy vegetables) (78). The WHO approved the recommendation in 2006, that for reproductive-aged women the daily intake of folic acid should be 400 ug from contemplating pregnancy until 12 weeks of gestation (80). No consistent agreement currently exists on the need to maintain this high folic acid intake during the entire pregnancy period.

Low maternal B12 levels have been associated with the development of offspring obesity, hypertension, alteration in lipid metabolism, insulin resistance, and metabolic syndrome (81–83). The natural sources of vitamin B12 are for example eggs, meat, milk, and seafood. No international consensus exists for the definition of B12 deficiency in pregnancy until now, except for a few agreements that it should be between 120 and 220 pmol/L (84).

Evidence for the association between the availability of methyl donors throughout pregnancy and the development of obesity and related metabolic changes in the progeny caused by alterations in DNA methylation pattern originates from investigations of the yellow Avy agouti mice. In yellow Avy agouti mice, maternal diets low in methyl donors (vitamin B12, folic acid, choline, betaine, and methionine) resulted in hypomethylation of the agouti gene (33) which predisposed the progeny to increased somatic growth, obesity, hyperinsulinemia, and diabetes (85). During critical stages of early embryonic development, an inadequate maternal one-carbon cycle metabolism nutrient intake has been related to DNA hypomethylation at the agouti locus (86), predisposing to adult obesity and diabetes (87).

In rats, a maternal methyl-group donor supplemented diet decreased the leptin concentration of the offspring and caused decreased methylation of the leptin gene promoter. Female and male offspring exhibited altered post-natal growth, but the long-term body weight gain increased only in males (62).

A study in zebrafish presented that a maternal one-carbon cycle metabolism nutrient-deficient diet (choline, methionine, B9, B12, and B6) affected the hepatic methylation of DNA and gene expression of lipogenesis resulting in the accumulation of lipids in the liver of the progeny (only male offspring were investigated) (66).

In sheep models, the restriction of folate, B12, and methionine during the conception stage of the mother resulted in elevated blood pressure and, increased insulin resistance in its (male and female) offspring (61). Furthermore, increased adiposity and male-specific demethylation of affected loci were detected in the adult male progeny (61).

In the human study of McCullough et al. (Newborn Epigenetics Study) the associations between maternal homocysteine concentrations, vitamins B12, and B6, on progeny’s birth weight and weight gain, and DNA methylation at 4 DMR-s (PLAGL1, SGCE/PEG10, MEG3, and H19) was investigated. The progeny (50% males) of the mothers in the lowest quartile of B12 had higher early-life weight gain in comparison to the progeny of the mothers in the highest quartile of B12. Furthermore, higher early-life weight gain was observed among the male progeny of the mothers in the highest quartile of maternal B6. MEG3 DMR methylation was positively associated with maternal B6 concentrations (67).

Recent studies showed an association between altered DNA methylation patterns of genes (most investigated genes: IGF2, H19, LINE-1, ZAC1, MEG3) and maternal intake of methyl-group donors on offspring birth weight (60). In these studies, the effect on progeny’s long-term health was not investigated, despite the well-described associations between adult chronic diseases and lower birth weight (88). The results suggest that folate deficiency of the mother during pregnancy may predispose the offspring to develop non-communicable diseases in later life. This could be partly caused by low fetal growth mediated by epigenetic DNA modification in certain genes.

In summary, animal and human studies suggest that methyl-group donor intake in the prenatal period and pregnancy and lactation is associated with the development of obesity and related metabolic changes (dyslipidemia, glucose homeostasis, insulin resistance, decreased leptin concentration, and elevated blood pressure) by changing the methylation pattern of several genes (POMC, LEP, Map2k4, Irs2, Ephb2, Fgf21) playing an important role in the regulation of energy balance, food intake, insulin resistance, and hypertension.

## 4 Discussion

Recently, several studies showed an association between different nutrient intake and epigenetic modifications that alter gene expression and may result in an increased susceptibility to develop obesity and related metabolic changes (reviewed by Milagro 2012 and by Cai et al., 2021) (89, 90). The joint effect of several types of nutrients is however difficult to interpret, therefore it is necessary to investigate each ingredient separately.

**Figure 1:**
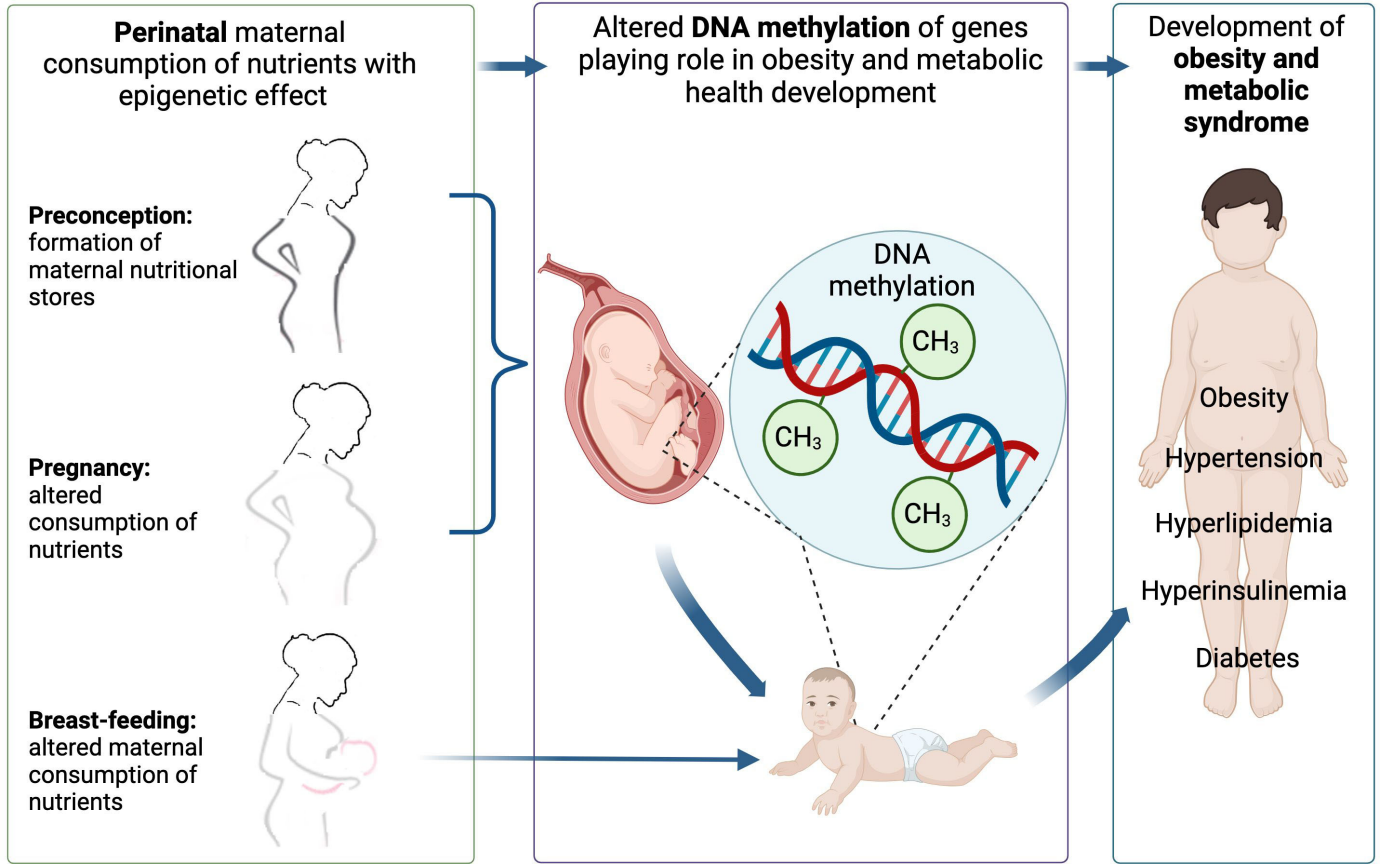
The association between perinatal maternal dietary factors and DNA methylation on the development of offspring obesity. Image was created with BioRender.com.

**Figure 2:**
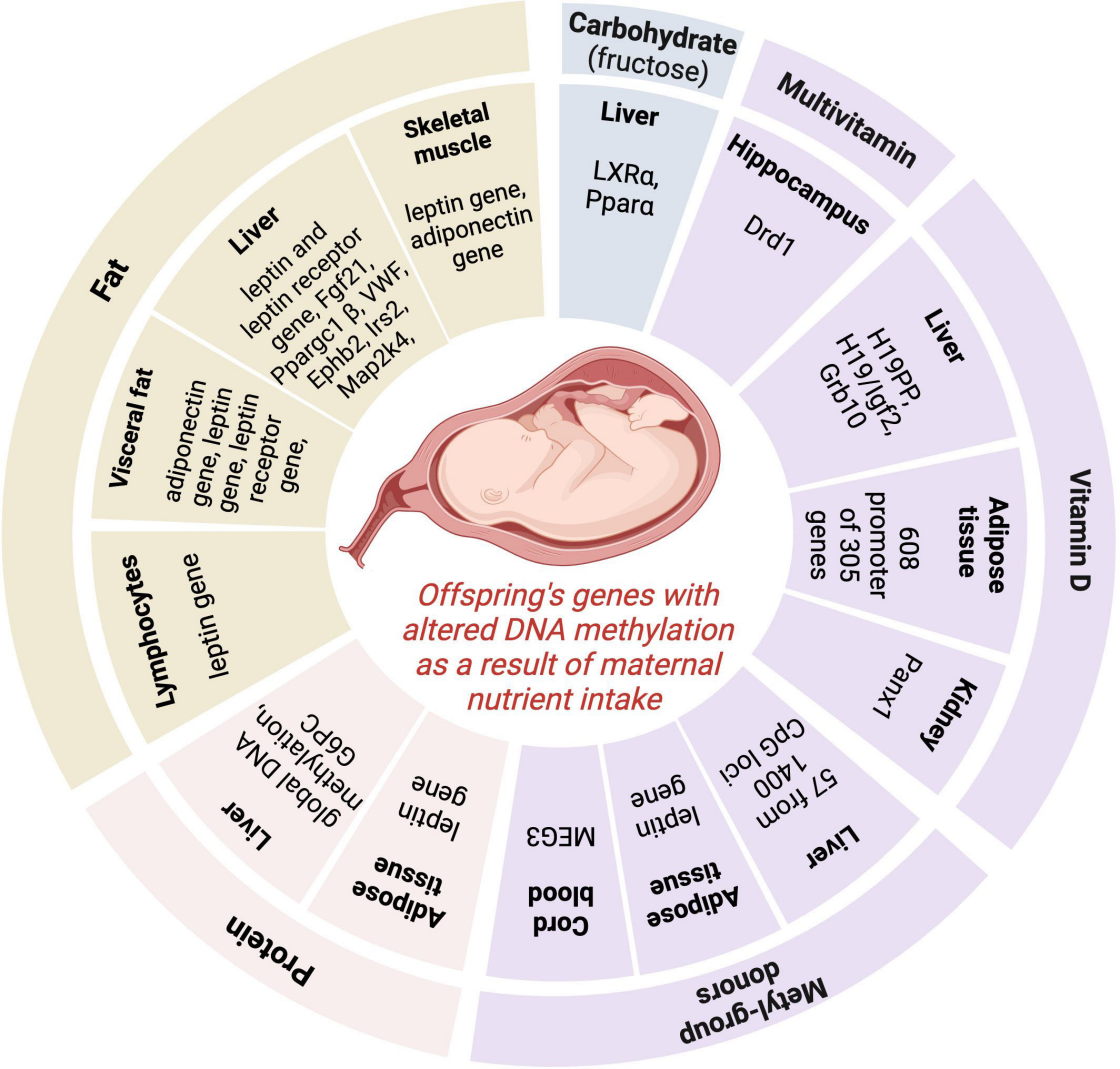
The summary of genes where altered DNA methylation was described in the offspring as a result of modified individual nutrient intake of the mother during pregnancy in the different tissues*. Pparα*, Peroxisome proliferator-activated receptor alpha; *LXRα*, Liver X receptor alpha; *G6PC*, glucose-6-phosphatase gene; *Fgf21,* Fibroblast growth factor 21; *Ppargc1β,* Peroxisome proliferator-activated receptor gamma coactivator 1-beta; *VWF,* von Willebrand factor*; Ephb2,* Ephrin type-B receptor 2*; Irs2*, Insulin Receptor Substrate 2; *Map2k4*, mitogen-activated protein kinase 4; *Drd1*, Dopamine receptor D_1_ *; Igf2*, Insulin-like growth factor 2; *Grb10*, Growth factor receptor-bound protein 10*; MEG3*, Maternally Expressed 3. Image was created with BioRender.com.

Maternal consumption of different nutrients throughout pregnancy could be associated with the development of obesity and related metabolic changes in the offspring via alteration in the DNA methylation pattern of different genes (**Figure 1**). The number of investigations where the individual nutrient intake of the mother during gestation (or gestation + lactation or gestation + just before gestation or gestation + just before gestation + lactation), the DNA methylation pattern of the offspring, and measurements included obesity development or related metabolic changes in the offspring’s later life were investigated in the same study are scarce. With our literature search, we found an association between alterations in the DNA methylation pattern of different genes caused by maternal under- or overnutrition of several nutrients (protein, fructose, fat, vitamin D, methyl-group donor nutrients) during the prenatal period, pregnancy, and lactation period and the development of obesity or related metabolic changes (glucose, insulin, lipid levels, blood pressure, non-alcoholic fatty liver disease) in the offspring. The summary of genes where altered DNA methylation was described in the offspring as a result of altered individual nutrient intake of the mother during pregnancy in the different tissues is presented in **Figure 2**.

We can see a big heterogeneity of identified genes (playing a role in different pathways of metabolism) having altered DNA methylation patterns in the offspring caused by changed individual nutrient intake of the mater during pregnancy. Only in the case of maternal fat intake can we detect a focus: the DNA alteration of the leptin gene was shown in several tissues, highlighting the role of this pathway in the epigenetic role of fat intake during pregnancy on the development of obesity in offspring. However, it is difficult to draw conclusions from these results and find emerging patterns of the epigenetic effect of each nutrient in obesity development because of several reasons: 1/the low number of complex investigations (nutrient intake during pregnancy + DNA methylation pattern of off-spring + measurements of obesity and/or related metabolic changes in the same study); 2/differences in DNA methylation assessment (investigation of global DNA methylation pattern or DNA methylation pattern of target genes); 3/the heterogeneity of cells studied (blood, hepatic cells, kidney cells, aorta cells, sperm, arcuate nucleus); 4/differences in the timing of the nutritional intervention (during gestation or just before gestation + gestation or just before gestation + gestation + lactation, or gestation + lactation or just before gestation); 5/difference in the gender of the offsprings included in the study (investigating only male- or only female offsprings or male + female offsprings together). Furthermore, evidence has shown that epigenetic gene regulation is rarely caused by 1 epigenetic modification alone; most regulation involves synergy among a variety of epigenetic modifications, in which histone and DNA methylation, are highly linked (90, 91). Synergies among different epigenetic modifications in the epigenetic regulation of early embryo development are summarized in the review of Cai et al. (90). As, in the present study, our aim was to summarize the results of those studies where the effect of alterations in DNA methylation caused by maternal nutrient intake was investigated, we can’t exclude that the results shown in our study are influenced by synergies among different epigenetic modifications.

Although an increasing body of evidence (using mainly *in vitro* models) highlights that individual nutrient availability, during the pre-implantation embryo development (the critical time period of the demethylation and remethylation), can trigger lasting epigenetic alterations in the offspring (92) The literature search was not able to detect (*in utero*) studies that investigated the complex association between alteration of DNA methylation pattern of the offspring caused by individual nutrient intake of the mother focusing exclusively on the pre-implantation period and the development of obesity or related metabolic changes in offspring’s later life. In studies investigating the associations between the individual carbohydrate and protein intake of the mother and alteration of the DNA methylation pattern of the offspring, the mothers were supplemented during whole gestation or whole gestation + lactation period. In the studies investigating the associations between the individual fat intake of the mother and DNA methylation pattern of the offspring, the timing of interventions was the following: 1/mothers were supplemented 58 days or 12 weeks before pregnancy, 2/two weeks before pregnancy + during pregnancy, 3/4 weeks before pregnancy+ pregnancy + lactation and 4/during whole gestation. Studies investigating the associations between the individual vitamin or methyl-group donor intake of the mother and alteration of DNA methylation pattern of the offspring the mothers were supplemented before pregnancy (5 weeks or 21-28 days) + during pregnancy + lactation or before pregnancy (8 weeks) until 6 days after conception or before pregnancy (4 weeks or 10 days) + during pregnancy. The above-described heterogeneity of the studies does not allow us to draw conclusions about the associations between the timing of nutrient intake of the mother and the resulting alterations in the DNA methylation pattern of the offspring.

Several other factors, for example, the epigenetic effect of the maternal conditions (e.g. maternal gut microbiome, obesity, diabetes) (93–97) or other maternal lifestyle factors (e.g. exposure to tobacco and alcohol, endocrine disruptors, stress) (93) could influence epigenetic changes and consequently, our results, that makes the interpretation of the findings even more difficult and emphasizes the importance of the multi-omics studies in this field.

The results were mainly drawn from animal experiments. Studies conducted in humans are very limited in this field and the follow-up of the examined offspring does not last into adulthood, which does not allow us to draw conclusions for later life.

The summary of the results of our narrative literature review demonstrates mounting evidence that the offspring’s DNA methylation pattern may be affected by the altered maternal intake of individual nutrients (fructose, fat, protein, vitamins, methyl-group donors) during pregnancy leading to the development of obesity and related metabolic changes in the offspring. For some of the investigated nutrients, like nutrients involved in the one-carbon metabolism, the exact role in DNA methylation is clearly described, while for other nutrients (LCPUFA, Vitamin D, fructose) the clear background of the mechanism of how the nutrients can alter DNA methylation pattern remains an important topic of investigation.

The main conclusion of the present review is that more studies are needed to better understand the associations between individual nutrient availability during pregnancy, alteration in DNA methylation, and the development of offspring’s obesity and related metabolic changes in later life.

## Author contributions

SB: Conceptualization, Data curation, Investigation, Writing – original draft. IC: Investigation, Methodology, Visualization, Writing – original draft. RF: Data curation, Investigation, Visualization, Writing – original draft. RV: Investigation, Methodology, Writing – original draft. SF: Conceptualization, Investigation, Methodology, Writing – original draft. TE: Conceptualization, Supervision, Writing – original draft. DM: Conceptualization, Investigation, Methodology, Supervision, Writing – review & editing.
